# Single vector non-leaky gene expression system for *Drosophila melanogaster*

**DOI:** 10.1038/s41598-017-07282-w

**Published:** 2017-07-31

**Authors:** Arslan Akmammedov, Marco Geigges, Renato Paro

**Affiliations:** 1 Department of Biosystems Science and Engineering, Federal Institute of Technology Zürich, Mattenstrasse 26, 4058 Basel, Switzerland; 2 Faculty of Science, University of Basel, Klingelbergstrasse 50, 4056 Basel, Switzerland

## Abstract

An ideal transgenic gene expression system is inducible, non-leaky, and well tolerated by the target organism. While the former has been satisfactorily realized, leakiness and heavy physiological burden imposed by the existing systems are still prominent hurdles in their successful implementation. Here we describe a new system for non-leaky expression of transgenes in *Drosophila*. PRExpress is based on a single transgenic construct built from endogenous components, the inducible *hsp70* promoter and a multimerized copy of a Polycomb response element (PRE) controlled by epigenetic chromatin regulators of the Polycomb group. We show that this system is non-leaky, rapidly and strongly inducible, and reversible. To make the application of PRExpress user-friendly, we deliver the construct via site-specific integration.

## Introduction

Inducible gene expression systems (IGES) have made a substantial contribution to basic research and applied sciences. In their principal configurations, IGES consist of two transgenic components and an inducer (e.g. doxycycline). One of the transgenes contains a gene of interest (GOI) under the control of a regulatable promoter, while the other constitutively expresses a heterologous transactivator. Thus, in the presence of the inducer, the transactivator can bind to the regulatable promoter and activate transcription of the GOI. In more advanced IGES, a third transgene carrying a transcriptional repressor is introduced to reduce leaky expression of the GOI.

Table 1 lists popular examples of gene expression systems used for temporal control of GOI, in comparison to the PRExpress system described in this study. TARGET and GeneSwitch systems are based on the GAL4-UAS system^5^, which consists of yeast *Upstream Activating Sequence (UAS)* and the trans-activating GAL4 transcription factor (TF). GAL80, another yeast TF, binds as a dimer to GAL4 and prevents recruitment of the Pol II transcription machinery, thereby repressing activation of *UAS* promoters^6^. GAL80ts is a temperature-sensitive allele inactive at 30 °C. Thus, in TARGET, a temperature shift from permissive 19 °C to restrictive 30 °C inactivates GAL80ts and allows induction of the GOI. GeneSwitch, on the other hand, employs the GLp65 fusion protein containing the GAL4 DNA binding domain, a mutated progesterone receptor-ligand binding domain and a part of the human p65 protein activation domain^7^. The binding of GLp65 to *UAS* is sensitive to RU486, an analog of progesterone. Feeding flies food containing RU486 induces the expression of the GOI in GeneSwitch. The Q system is conceptually similar to GAL4-UAS and is based on components from *Neurospora*. QF is a TF that activates *QUAS* promoter. This activation is inhibited by QS, a repressor. The QS-mediated inhibition is relieved upon feeding of quinic acid to flies thereby inducing the expression of the GOI. Tetracycline inducible systems are arguably the most frequently used gene expression systems across different model organisms and tissue cultures. In the Tet-On system, the affinity of reverse tetracycline transactivator (rtTA) to the *tetO* sequence is sensitive to the presence of doxycycline, a derivative of tetracycline^8^. Similar to GeneSwitch and Q system, Tet-On flies are fed doxycycline-containing food to induce expression of the GOI. For comparison, the PRExpress system described in this study is also included in Table 1.

**Table 1:** Systems for temporal gene expression control in *Drosophila*.

System	TARGET^1^	GeneSwitch^2^	Q system^3^	Tet-On^4^	PRExpress
Promoter	*UAS*	*UAS*	*QUAS*	*tetO*	*poly-PRE-hsp70*
Activator	GAL4	GLp65	QF	rtTA	Heat-shock response system
Repressor	GAL80ts		QS		PcG system
Inducer	Shift from 19 °C to 30 °C	RU486	Quinic acid	Doxycycline	Shift from 25 °C to 37 °C
Reporter	GFP	GFP	GFP, tdTomato	LacZ, luciferase	LacZ
Stage	A	L	L, A	E, L, A	E, L, A
Kinetics	On – 12 h, Off_50%_ – 15 h	On – 21 h, Off – 72 h	On – 24 h, Off – N/A	On – 3 h, Off – N/A	On – 0.5 h, Off_2%_ – 2 h
Leakiness	Brain (A)	Whole fly extract (L)	Brain (A)	Whole fly extract (A)	No leakiness*

A – adult, L – larva, E – embryo. * – see results and discussion.

Among the biggest advantages of the aforementioned systems is the ability to control gene induction not only temporally but also spatially. Spatial control is achieved through the use of drivers with tissue-specific expression of heterologous transactivators GAL4, GLp65, QF, and rtTA. There are, however, some drawbacks as well. Induction kinetics of the GOI in these systems is rather slow: time until full induction ranges from 3 to 24 hours and re-silencing is achieved within 15 to 72 hours. In case of GeneSwitch, Q system, and Tet-On, this can be due to the necessity of feeding the inducer to flies. This aspect also restricts the induction of the GOI to the feeding stages of the organism: larva and adult. The heterologous nature of transactivators is frequently assumed to be synonymous with no impact on endogenous cellular processes. This is, however, not the case and ubiquitous expression of GAL4 and QF is toxic to flies^3^ and even sub-toxic levels can have significant phenotypic effects^9–11^.

Leakiness, the uncontrolled expression of the GOI, is especially problematic as it confounds the interpretation of results and can lead to erroneous conclusions that can require substantial efforts to rectify^12, 13^. As noted in Table 1, leakiness was observed with each system described. One caveat is that these studies mostly focused on a single developmental stage or only on certain tissues within that stage. Therefore, little is known about leakiness at earlier or later stages and in the rest of the organism. Leakiness can be caused by the activation of a regulatable promoter by endogenous transcription factors in the absence of the heterologous transactivator and inducer^14–16^, by heterologous transactivators in the absence of the inducer^4, 17, 18^, or by trapping a near-by endogenous regulatory element^5^. The latter effect in combination with random transgene integration strategy (RTIS) results in independent transgenic lines. These lines carry the same transgene integrated at different genomic locations with distinct transgene induction characteristics: weak or strong induction, low or high leakiness. A dramatic example of this variability was observed in a transgenic mouse model when fluorescent protein reporters were placed under the control of identical *thy1* gene regulatory elements and randomly integrated into the genome. Remarkably, each of the 25 independently generated lines displayed a unique expression pattern^19^. Similarly, flies carrying the same construct containing the *mini-white* marker gene integrated into different genomic loci exhibited eye color ranging from light orange to red^20^. In addition to variability introduced by random integration of the GOI, drivers influence leakiness of the system. Scialo and colleagues, for example, used the GeneSwitch system and reported that the use of a stronger *tubulin-Gene-Switch* driver resulted in higher leakiness of a *UAS-lacZ* reporter compared to a weaker *daughterless-Gene-Switch* driver in the absence of the inducer^18^. Poirier and colleagues also using the GeneSwitch system, reported that leakiness of a *UAS-lacZ* reporter depended on the driver, developmental stage, tissue analyzed, and sex of the fly^17^.

The promoter of the *hsp70* gene in *Drosophila* is an endogenous, inducible promoter. The induction of the *hsp70* promoter depends on the duration and temperature of heat-shock^21^. Additionally, it has been shown to be active and induce expression upon heat-shock in plant^22^, yeast^23^, frog^24^, mouse^25^, monkey^26^, and human^27^ cells. The induction of the *hsp70* promoter is fast and reversible^28^ and dependent on stress-induced oligomerization and binding of heat-shock factor (HSF)^29^. While they lack the toxicity associated with the expression of heterologous transactivators and the administration of the inducer, widespread use of endogenous inducible promoters is hampered by their generally high leakiness^30, 31^.

Polycomb group (PcG) proteins together with counteracting Trithorax group (TrxG) proteins are responsible for stable and heritable maintenance of specific gene-expression patterns necessary for cell-lineage identity. Both TrxG and PcG proteins are highly conserved between flies and mammals. In early *Drosophila* embryos, spatial expression patterns of *HOX* genes are established by gap and pair-rule transcription factors. Later in embryonic development, these gap and pair-rule regulators are no longer expressed and TrxG and PcG proteins take over the roles of maintaining expression and repression of homeotic genes, respectively^32^. The repression is achieved through recruitment of PcG proteins to PREs located in vicinity of PcG target genes and deposition of the H3K27me3 silencing mark^33^. The maintenance of expression, on the other hand, requires binding of TrxG proteins to PREs and deposition of the H3K4me3 mark^34^. The homeotic gene *Ultrabithorax (Ubx)* is a PcG-target gene and is expressed in parasegments 5 and 6 and repressed in parasegments 1–4 and 7–14 of *Drosophila* embryo. A regulatory region of *Ubx* placed upstream of a *lacZ* reporter gene and ectopically integrated into the *Drosophila* genome has been shown to repress the reporter in parasegments 1–5. This repression was shown to be dependent on PcG proteins and the *bithoraxoid (bxd)* PRE^35^. The *bxd* PRE was also shown to function in combination with enhancers from other genes and resulted in tissue-specific repression of the reporter that depended on activity of a given enhancer in early embryogenesis^36^.

Here we describe a novel gene expression system, PRExpress, based on the *hsp70* promoter and an octamerized fragment of the *bxd* PRE. We show that PRExpress is an inducible, reversible, and non-leaky gene expression system. To eliminate variation inherent with RTIS, we use site-specific integration to deliver PRExpress and evaluate integration sites on the 2^nd^ and 3^rd^ chromosome.

## Results

### Generation of PRExpress

**Figure 1:**
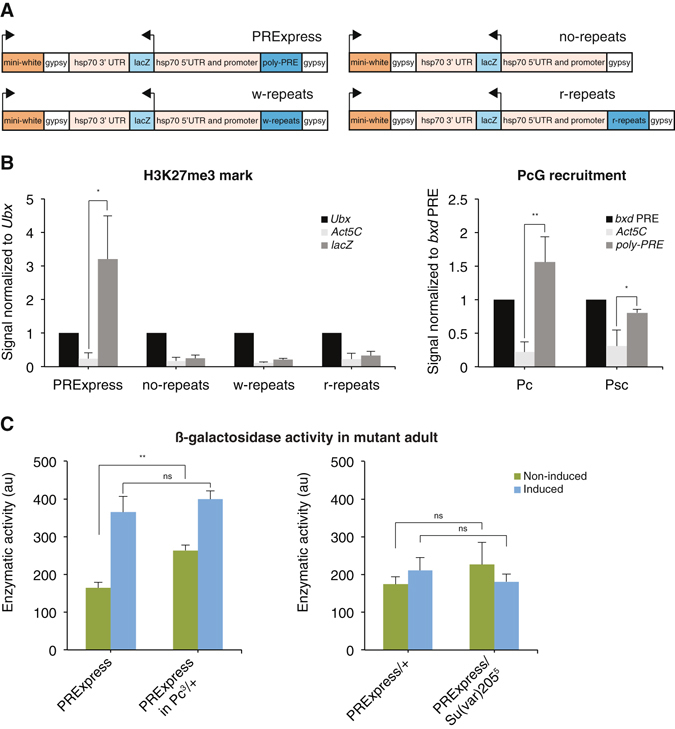
PRExpress in *Drosophila*. (**A**) Schematic view of transgenic constructs. In PRExpress, the *lacZ* coding sequence was placed under the control of *hsp70* regulatory sequences. The *poly-PRE* sequence consisting of 8 repeats of a 198-bp fragment of the *bxd* PRE was placed upstream of the *hsp70* promoter. This transgene was placed in the *gypsy* insulator bracket to reduce the potential repression of the *mini-white* marker gene. No-repeats control is similar to PRExpress but without any repeat sequences upstream of the promoter. In w-repeats and r-repeats controls, the *poly-PRE* sequence was replaced by 8 repeats of 198-bp fragments of the *white* gene and a random sequence, respectively. (**B**, left**)** Histone H3K27me3 mark at the reporter gene in PRExpress and no-repeats, w-repeats, and r-repeats control embryos. Chromatin immunoprecipitation (ChIP) results were normalized to *Ubx* gene values, a positive control. *Act5C* was a negative control. (**B**, right) PcG recruitment at *poly-PRE*. Anti-Pc and anti-Psc ChIPs were normalized to *bxd* PRE, a positive control. *Act5C* was a negative control. (**C**) De-repression of PRExpress in heterozygous PcG and heterochromatin protein mutant backgrounds measured by β-galactosidase activity assay of whole adult protein extracts. The reporter was de-repressed in non-induced Pc^3^ mutant background but not in Su(var)205^5^ mutant background, affecting heterochromatin silencing. NS – *P* > 0.05, *P ≤ 0.05, **P < 0.01, n = 3.

The *E. coli* β-galactosidase gene, *lacZ*, was used as the reporter gene in this study. The reporter was placed under the control of regulatory sequences of the *hsp70* gene to allow ubiquitous heat-inducible expression (Fig. 1A). A 198-bp fragment of the *bxd* PRE, BP, was shown previously to retain PRE activity *in vivo* ^37^. To render the *hsp70* promoter non-leaky in the absence of induction, eight copies of the BP fragment, *poly-PRE*, were cloned upstream of the *hsp70-lacZ* expression cassette. The reporter gene together with *poly-PRE* was placed within a *gypsy* insulator bracket. *gypsy* insulator sequences can efficiently block PRE-mediated silencing^38^ and were cloned to reduce potential repression of the mini-white marker gene^37^. We also generated three control lines: no-repeats line with no repeat sequences upstream of the *hsp70* promoter, and w-repeats and r-repeats lines with eight repeats of a 198-bp fragment of the 2^nd^ exon of the *white* gene and a 198-bp fragment of a random sequence, respectively. The w-repeats and r-repeats lines were generated to rule out the possibility that repeated sequences alone, irrespective of the identity of the sequence, result in the repression of the reporter. PRExpress as well as control constructs were delivered to the 2^nd^ chromosome via ϕC31-mediated integration^20^. First, we tested whether *poly-PRE* recruited PcG proteins and resulted in the deposition of the H3K27me3 histone mark in the reporter gene. Left bar graph of Fig. 1B shows results of a chromatin immunoprecipitation (ChIP) experiment performed on PRExpress, no-repeats, w-repeats, and r-repeats embryos collected overnight (0–16 hours old). The H3K27me3 mark at the *lacZ* reporter showed higher enrichment compared to the *Ubx* gene, a positive control, and significantly higher enrichment compared to the negative control, *Act5C* gene, in PRExpress embryos. In no-repeats, w-repeats, and r-repeats embryos the enrichment of this mark at the *lacZ* reporter was at the background level. Right panel of Fig. 1B shows recruitment of PcG proteins Polycomb (Pc) and Posterior Sex Combs (Psc) to *poly-PRE* in the PRExpress line. Primers for amplification of *poly-PRE* and the *bxd* PRE were designed to amplify BP fragment specifically from either *poly-PRE* in the transgene or its endogenous location in the *bxd* PRE. Enrichment of Pc and Psc at *poly-PRE* was significantly higher compared to the negative control, *Act5C*, and similar to the *bxd* PRE, the positive control. Next, to test whether the repression of the reporter was dependent on the PcG system or heterochromatinization of the transgene, PRExpress was introduced in Pc^3^ and Su(var)205^5^ mutant backgrounds. The Pc^3^ allele is homozygous lethal, while heterozygotes are viable with males showing homeotic transformations of the second and the third leg^39^. The Su(var)205^5^ allele is also homozygous lethal, while heterozygotes show a suppression of the position effect variegation caused by In(1)w^m4^ chromosomal inversion due to compromised ability to form heterochromatin^40^. Figure 1C shows results of β-galactosidase activity assays of whole fly protein extracts. This assay permits quantitative assessment of the amount of LacZ protein in the sample. In this assay, a non-fluorescent substrate, MUG, is enzymatically converted by β-galactosidase into a fluorescent product, 4-MU. Thus, the intensity of 4-MU fluorescence is proportional to the amount of LacZ protein^41^. The enzymatic activity of protein extract was higher in the presence of the Pc^3^ allele in the non-induced condition, indicating the de-repression of the *lacZ* reporter in the PcG mutant background. Upon induction, there was no difference between the Pc^3^ mutant and non-mutant backgrounds. The presence of the Su(var)205^5^ allele did not lead to any significant de-repression of the *lacZ* reporter in either the induced or non-induced condition. In addition, the *lacZ* gene was not significantly upregulated upon heat-shock in the Su(var)205^5^ experiment in either mutant or non-mutant background. Since both PRExpress and the Su(var)205^5^ allele are located on the 2^nd^ chromosome, PRExpress was heterozygous in this experiment. This suggested that a single copy of PRExpress in the Su(var)205^5^ experiment was not sufficient to produce 2-fold induction seen in homozygous PRExpress adults (Figs 1C, 3B and C). Together these results suggest that the synthetic *poly-PRE* acts similarly as endogenous PRE sequences.

### PRExpress performance in embryos

**Figure 2:**
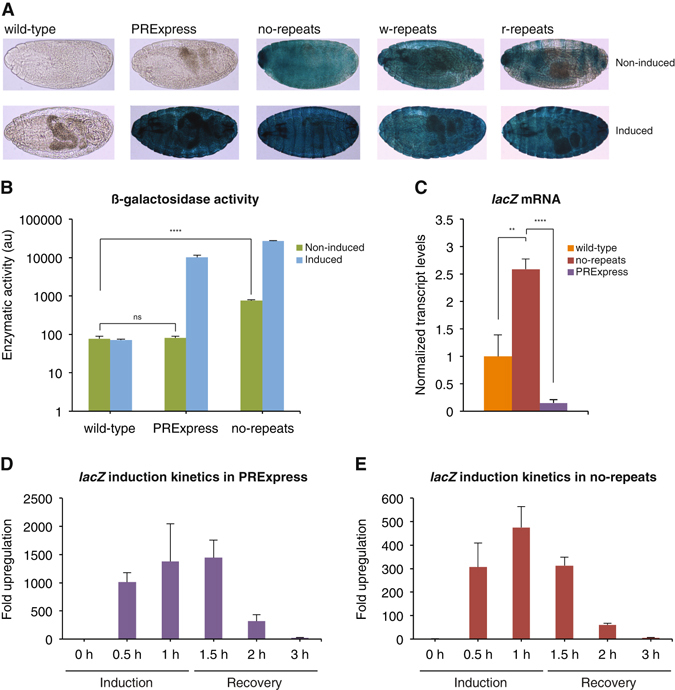
PRExpress in embryos. (**A**) X-gal staining of non-induced and induced embryos. (**B**) β-galactosidase activity of protein extracts from wild-type, PRExpress, and no-repeats embryos. PRExpress showed no evidence of leakiness, while no-repeats control had 10-fold higher activity compared to wild-type. Statistical significance of comparisons between non-induced and induced wild-type, PRExpress, and no-repeats samples is given in Supplementary Table 1. (**C**) Non-induced lacZ transcript levels in wild-type, no-repeats, and PRExpress embryos. At the mRNA level, no-repeats control showed higher levels of *lacZ* mRNA compared to wild-type and PRExpress, while PRExpress showed no evidence of leakiness. The values for *lacZ* mRNA were first normalized to *RpL32* and then to the average of normalized wild-type values. (**D**,**E**) Kinetics of *lacZ* mRNA induction in PRExpress and no-repeats embryos. Both lines showed rapid and reversible induction of the reporter. The embryos were induced for 1 hour and then allowed to recover for up to 2 hours. The values for *lacZ* mRNA were normalized to *RpL32* and the 0 h time point values. NS – *P* > 0.05, *P ≤ 0.05, **P < 0.01, ****P < 0.0001, n = 3.

Expression of *lacZ* can also be evaluated qualitatively by X-gal staining. In this assay, the colorless soluble X-gal substrate is converted by β-galactosidase into an insoluble blue product. Thus, the presence of the blue-colored product is indicative of the β-galactosidase activity^42, 43^. Preliminary experiments were conducted to identify the staining period after which wild-type samples started to develop blue staining. Wild-type samples stained for more than 4 hours developed blue staining complicating the interpretation of results. Hence, to eliminate this problem, a 4-hour staining period was used to detect leaky expression throughout this study. Since a completely silent reporter gene would not produce any mRNA or protein and would be similar to a fly line without the reporter gene, Oregon-R-P2 wild-type line was used as negative control in all experiments. Induction was defined in this study as an incubation of fly embryos, larvae, and adults for 1 hour at 37 °C followed by a 30-minute recovery period at 25 °C after which *lacZ* expression was analyzed. The survival rate of embryos to adulthood after the induction was 85–89% for wild-type, PRExpress, and no-repeats compared to non-induced embryos (Supplementary Fig. 1). Figure 2A shows results of X-gal staining of non-induced and induced embryos collected overnight (0–16 hours). Instead of focusing on a specific embryonic stage, we evaluated leakiness as a feature throughout different embryonic stages available in overnight collections and showing characteristic staining patterns. In the non-induced state, PRExpress and wild-type embryos showed no staining, while the control lines no-repeats, w-repeats, and r-repeats exhibited blue staining, indicating leaky expression of the reporter. Upon induction, all lines except wild-type showed strong staining. To quantitatively compare the amount of the LacZ protein in wild-type, PRExpress, and no-repeats lines, we evaluated the β-galactosidase activity of whole embryo protein extracts. In the non-induced state, there was no difference in the β-galactosidase activity between wild-type and PRExpress embryos, while a 10-fold higher enzymatic activity was observed in no-repeats embryos confirming leakiness observed with X-gal staining (Fig. 2B). After heat-shock, β-galactosidase activity of PRExpress and no-repeats embryos increased 124-fold and 36-fold, respectively. However, absolute levels of β-galactosidase activity in the induced state were 3-fold higher in the no-repeats embryos compared to PRExpress embryos. Similar to protein levels, *lacZ* mRNA levels in the non-induced state were higher in no-repeats embryos compared to wild-type and PRExpress embryos, while PRExpress embryos showed no evidence of leakiness (Fig. 2C). Since no *lacZ* gene is present in wild-type embryos, the signal shown for wild-type in Fig. 2C represents background. At background levels, quantitative RT-PCR values fluctuate^44^ and a lower PRExpress value compared to wild-type is not unexpected. We also evaluated kinetics of induction by measuring *lacZ* mRNA levels during induction and recovery period (Fig. 2D). 30 minutes of induction was sufficient to raise *lacZ* mRNA levels 1,000-fold in PRExpress embryos and, after two hours of recovery, the amount of the *lacZ* mRNA present was less than 2% of the induced level (1.5 h). The induction kinetics of the *lacZ* mRNA in no-repeats control (Fig. 2E) was similar to the PRExpress line, with strong induction after 30 minutes of heat-shock and rapid decrease of *lacZ* mRNA during the recovery period. However, while the fold upregulation of *lacZ* mRNA was lower in no-repeats compared to PRExpress embryos, the absolute induced mRNA levels were at least 4-fold higher in no-repeats compared to PRExpress embryos (Supplementary Table 2). As a negative control, an induction kinetics experiment was performed on wild-type embryos and results are shown in Supplementary Fig. 2. In the absence of the *lacZ* reporter gene, wild-type values fluctuate at the background level.

### PRExpress in larva, adult, and 3^rd^ chromosome integration site

**Figure 3:**
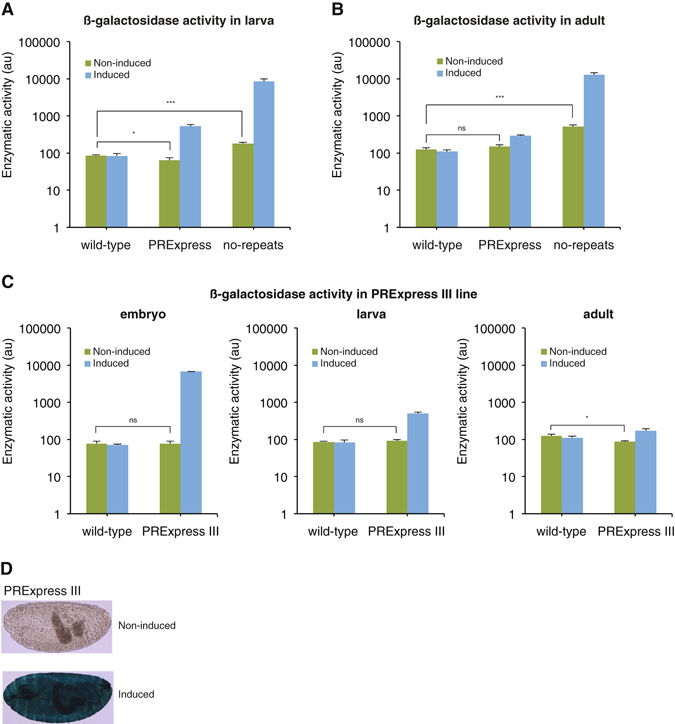
PRExpress in larva, adult, and at 3^rd^ chromosome integration site. (**A**) β-galactosidase activity of larval protein extracts. In the non-induced state, no-repeats control showed 2-fold higher activity levels than wild-type, while PRExpress showed lower levels than wild-type. Upon induction, PRExpress and no-repeats showed increased activity levels, with activity of no-repeats extract higher than PRExpress. (**B**) β-galactosidase activity of adult fly protein extracts. In adult flies, no-repeats showed 4-fold higher activity in the non-induced state, while PRExpress was not significantly different from wild-type. In induced state, no-repeats showed a strong increase in activity, while PRExpress showed only 2-fold increase. (**C**) β-galactosidase activity of embryonic, larval, and adult fly whole protein extracts from wild-type and PRExpress III lines. There was no evidence of leakiness in the non-induced state. The β-galactosidase activity in PRExpress III adults was lower than in wild-type adults. Upon induction, PRExpress III line showed similar increases in activity as PRExpress line. (**D**) X-gal staining of non-induced and induced PRExpress III embryos. Statistical significance of comparisons between non-induced and induced wild-type, PRExpress, no-repeats, and PRExpress III samples in Fig. 3A–C is given in Supplementary Table 1. NS – *P* > 0.05, *P ≤ 0.05, **P < 0.01, ***P < 0.001, n = 3.

Next, we investigated leakiness and inducibility of the reporter gene in third instar wandering larvae and adults (3 days post eclosion). Similar to embryos, non-induced no-repeats control larvae and adults showed higher β-galactosidase activity than wild-type larvae and adults (2-fold and 4-fold, respectively), while PRExpress larval and adult extracts showed no evidence of leakiness (Fig. 3A and B). The activity of larval PRExpress protein extract was significantly lower than larval wild-type lysate in the non-induced state. Wild-type lysate value represents the background 4-MU fluorescence in this assay and fluctuations around this value are not unexpected. In addition, the differences between wild-type and PRExpress lysates was less than 1.5-fold and was assumed to be inconsequential (Fig. 3A). Upon induction, β-galactosidase activity of PRExpress in larvae and adults increased 8-fold and 2-fold, respectively, whereas activity of no-repeats increased 47-fold and 25-fold, respectively. This indicated reduction of *lacZ* induction in PRExpress with increasing developmental stage of the fly. The induction of *lacZ* in no-repeats flies was not dampened and the transgene remained strongly inducible at all stages tested. X-gal staining of various larval tissues indicated leakiness in the brain and salivary gland of no-repeats control larva (Supplementary Fig. 3), while PRExpress larva showed only minor leakiness in the brain.

So far, we described characteristics of PRExpress at different developmental stages for a 2^nd^ chromosome integration site. However, for PRExpress to be useful and compatible with a mutation or a transgene on any chromosome, an integration site on a different chromosome with similar characteristics needed to be described. To this end, we site-specifically introduced PRExpress on the 3^rd^ chromosome. The leakiness and induction profile of the line with the 3^rd^ chromosome integration, PRExpress III, were strikingly similar to the 2^nd^ chromosome integration line. The β-galactosidase activity of whole protein extracts showed induction in embryo, larva, and adult with increases in β-galactosidase activity of 88-fold, 6-fold, and 2-fold, respectively (Fig. 3C). There was also no evidence of leakiness in embryo, larva, and adult with β-galactosidase activity assay. The activity of adult PRExpress lysate was significantly lower than adult wild-type lysate in the non-induced state. In this case, the differences between wild-type and PRExpress lysates was also less than 1.5-fold and was assumed to be inconsequential. The induction of the reporter gene was also less efficient at the larval and adult stages in the PRExpress III line. X-gal staining of embryos confirmed absence of leaky expression in PRExpress III line (Fig. 3D). In X-gal staining of larval tissues, PRExpress III line showed similarity to the 2^nd^ chromosome integration, with the single observed difference between 2^nd^ and 3^rd^ chromosome integration sites of PRExpress being poor inducibility in the larval salivary gland of PRExpress III line (Supplementary Fig. 3). Since the only difference between these PRExpress lines was the integration site, poor inducibility in the larval salivary gland of PRExpress III was assumed to be due to the influence of the endogenous environment of the integration site.

### Comparison of PRExpress to the existing systems

**Figure 4:**
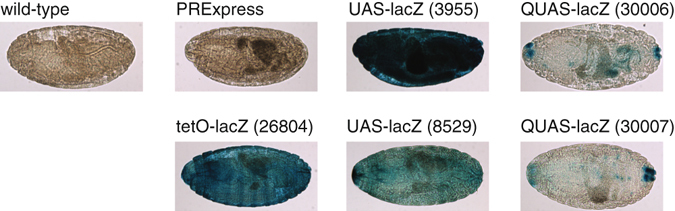
Leakiness of *lacZ* transgenes under *UAS*, *QUAS*, and *tetO* promoters. X-gal staining of embryos collected overnight (0–16 hours). Wild-type and PRExpress embryos showed no blue staining, whereas UAS, QUAS, and tetO lines showed blue staining indicating leakiness of their promoters.

Finally, we compared PRExpress to a selection of five publicly available *lacZ* reporter lines under the control of *UAS*, *QUAS*, and *tetO* promoters. Since the main advantage of any IGES is the ability to set the time point at which the GOI is induced, leakiness at the embryonic stage would complicate interpretation of results and compromise utility of the IGES at later stages as well^17, 45^. For this reason, we restricted our comparison to the embryonic stage of development (0–16 hours). Since there was no difference in reporter expression at the embryonic stage between the PRExpress and the PRExpress III lines, only the PRExpress line embryos were included in the comparison. Two of the eight *UAS-lacZ* lines (randomly selected), both of the two *QUAS-lacZ* lines, and the one *tetO-lacZ* line available at Bloomington Drosophila Stock Center (BDSC) were chosen. We selected lines that contained only the reporter transgene and no heterologous transactivators, thereby restricting the comparison to the assessment of the leakiness of regulatable promoters only, without addressing leakiness due to different drivers^17, 18^. Figure 4 shows results of the X-gal staining of embryos from the five fly lines together with wild-type and PRExpress embryos. The *UAS*, *QUAS*, and *tetO* promoters exhibited leakiness, while the PRExpress line was indistinguishable from the wild-type line. Both *QUAS* promoter lines exhibited a characteristic pattern of *lacZ* expression. *UAS-lacZ* line BDSC #3955 showed higher leakiness than line BDSC #8529, potentially indicating variation due to RTIS. The *tetO* promoter also showed high leakiness comparable to *UAS-lacZ* line BDSC #3955. The results of this comparison suggested that PRExpress exerts a tighter control of gene expression and, in this regard, is superior to the existing systems. These results also highlight that the same promoter integrated at different genomic locations can display different degrees of leakiness.

## Discussion

PREs are defined as cis-regulatory DNA elements that recruit PcG proteins to target genes. This recruitment subsequently results in H3K27me3 histone mark deposition and heritable silencing of the target gene in a PcG-depend manner (i.e. target gene de-repression in PcG mutant background)^46^. *poly-PRE* was able to recruit PcG proteins Pc and Psc and resulted in the establishment of H3K27me3 mark in the *lacZ* reporter gene. The presence of *poly-PRE* upstream of the promoter was shown to repress leaky transcription of the *hsp70* promoter and this repression depended on the PcG system and not heterochromatinization of the reporter. We also showed that the absence of leakiness with *poly-PRE* was dependent on the short PRE fragment within the repeats, as random fragment and the *white* gene fragment repeats failed to eliminate leaky expression.

Evaluation of leakiness in IGES is controversial due to differences in individual experimental setups. Reporters available for assessment of gene expression can be broadly divided into enzymatic and fluorescent protein reporters, with each group having their pros and cons. Fluorescent proteins enable *in vivo* visualization and tracking of gene expression over time without extensive sample processing. They are also readily amenable to flow cytometry for high-throughput single-cell readout experiments and to fluorescence-activated cell sorting for experiments analyzing sub-populations within a given pool of cells. The enzymatic reporters, on the other hand, require destruction of the cell and processing of the sample to “visualize” gene expression. Notwithstanding, enzymatic reporters are generally thought to be more sensitive than fluorescent reporters^47, 48^. Detection of fluorescent reporters is fundamentally limited at low expression levels, when the fluorescence of the reporter reaches the autofluorescence levels of the sample. In a strict comparison between eYFP and LacZ reporters in bacteria, the LacZ reporter was shown to be superior at low expression levels, whereas eYFP detection was limited by autofluorescence^49^. In addition, the detection limit of fluorescent proteins was shown to depend on the fluorescent protein, growth media, organism, and strain^50^. On the other hand, LacZ reporters permit both qualitative evaluation of expression pattern with X-gal staining and robust quantification of expression levels with β-galactosidase assay. Eukaryotic cells have endogenous β-galactosidase^51^, however, composition and pH of buffers can be adjusted to selectively increase LacZ reporter activity^52, 53^. Alternatively, quantitative RT-PCR can be used to evaluate *lacZ* expression without any interference from endogenous β-galactosidase.

We showed here that *UAS*, *QUAS*, and *tetO* promoters were leaky, whereas PRExpress was not. This is in agreement with previous studies showing leakiness of these promoters in different tissues and developmental stages^1–4, 16–18^. Any comparison of different IGES is necessarily arbitrary and selective since a comparison of all *regulatable-promoter-lacZ* lines in public and private stocks as well as evaluation of leakiness at all developmental stages and tissues is not practical. Use of RTIS to generate transgenic lines described in Table 1 and Fig. 4 is another confounding factor. RTIS reduce the amount of valuable information that can be extracted from reporter gene studies as any new integration site can have different induction characteristics and leakiness (see *UAS-lacZ* lines in Fig. 4). Flanking chromosomal sequences also influence PREs and, depending on the integration site, phenotypes can range from very weak repression to virtually complete silencing^54^. Therefore, the use of site-specific integration (e.g. ϕC31-mediated integration) is of great value in studies describing IGES.

One salient feature of PRExpress observed for both integration sites was the decreasing inducibility of the reporter with increasing developmental stage. Inducibility of *lacZ* was highest in embryos, lower in larvae, and minimal in adults. This feature is in agreement with what is known about the PcG system. SIR2 association with E(z), a component of Polycomb repressive complex 2, was reported to be restricted to post-embryonic stages of development and was suggested to increase fidelity of PcG silencing^55^. We have also previously shown increasing difficulty of activating a reporter gene controlled by PRE in larval stage compared to embryonic stage^56, 57^. Based on these observations, the chromatin of PcG-repressed genes in the embryo was suggested to be more plastic, and becomes progressively more committed as development proceeds^58^. This explains why the *hsp70* promoter in PRExpress was less inducible later in development, whereas the same promoter in no-repeats was highly inducible at all stages. This feature may limit applicability of PRExpress in its current configuration to embryonic and larval stages. Alternatively, fragments of weaker PREs or PREs that reduce expression rather than silence target genes can be used to maintain strong induction of the promoter at later developmental stages^59, 60^.

We also observed minor leakiness in larval brains of both PRExpress and PRExpress III lines with X-gal staining, whereas no leakiness was apparent in the β-galactosidase activity assay of larval protein extracts (Fig. 3A and C, Supplementary Fig. 3). This could be either due to differences in sensitivity of the two assays or an artifact of X-gal staining procedure. In β-galactosidase assay, live larvae are snap-frozen in liquid nitrogen preserving the cellular milieu at the moment of freezing. In X-gal staining, live larvae are dissected at room temperature in PBS and then transferred into the cross-linking solution containing formaldehyde. Thus, the larvae in X-gal staining procedure experience “the stress of dying” due to dissection and formaldehyde treatment^61^. Consequently, the outcome of the X-gal staining experiment in PRExpress lines would depend on relative rates of cross-linking reaction and stress-induced production of LacZ protein. RNA polymerase transcription at the 5′ end of the *hsp70* gene could be detected 30 seconds after heat-shock in *Drosophila* Schneider 2 cells^62^, whereas protein-protein and protein-DNA crosslinking is thought to occur within seconds to minutes^63–66^. Hence, on the issue of leakiness of PRExpress in the larval brain, our results are not conclusive and are open to interpretation.

Due to ubiquitous expression of HSF^67^ that drives transcription of the *hsp70* promoter, spatial control of gene expression with PRExpress is not feasible. However, PREs can repress transcription of both canonical PcG-target gene promoters and non-target gene promoters. *hsp26* ^54^, *miniwhite* ^68^, and *UAS* ^69^ promoters are among non-target gene promoters that were susceptible to PRE-mediated repression in flies. In mammalian cells, PREs were shown to repress viral *CMV* ^70^ and *SV40* ^71^ promoters. Thus, it might be possible to combine *poly-PRE* described here with *UAS*, *QUAS*, and *tetO* promoters and create a system that would allow a tight control of both temporal and spatial gene expression.

Immunogenicity^9^, global transcriptomic effects^10^, and emphysema-like changes^11^ have been associated with the expression of heterologous transactivators and repressors used in mammalian systems and for gene therapy applications. Use of non-leaky endogenous IGES, analogous to the one described here, can eliminate these undesired effects^30, 31, 72^. Based on high conservation of PcG system^73^ and recent discovery of mammalian PREs^74^, we hypothesize that synthesis of PREs and endogenous and exogenous regulatable promoters would usher a new generation of non-leaky gene expression systems.

In conclusion, we showed here that a simple single vector system based on endogenous regulatory regions of the *hsp70* gene and *bxd* PRE, PRExpress, achieves tightness of repression and rapid induction kinetics unmatched by currently available IGES. We report that PRExpress is non-leaky on 2^nd^ and 3^rd^ chromosomes, different developmental stages and irrespective of sex of the fly (both sexes were included in all of the experiments). We also used site-directed integration to deliver transgenes in this study. This allows prospective users integration of their GOI in the same locations to achieve similar induction characteristics and non-leakiness reported here.

## Methods

### Construction of transgenic vectors

To generate no-repeats control vector, promoter and 5′UTR region of *hsp70* gene, *lacZ* coding sequence, 3′UTR of *hsp70* gene, and *gypsy* insulators were cloned into pUASg.attB plasmid using standard restriction enzyme (RE) cloning. pUASg.attB plasmid was selected due to availability in the lab. Monomers of *poly-PRE*, *w-repeats*, and *r-repeats* (Supplementary Table 3) were octamerized using REs SpeI, NheI, and HindIII (NEB #R0133S, R0131S, and R0104S) and the strategy described previously^75^. Corresponding octamers were cloned into no-repeats vector to generate PRExpress, w-repeats, and r-repeats vectors. Annotated sequences of the plasmids are available in GenBank through the accession numbers MF058598 (PRExpress), MF058599 (no-repeats), MF058600 (w-repeats), MF058601 (r-repeats).

### Transgenic *Drosophila* lines

All fly lines were reared at 25 °C, in 60% humidity and at 13 hours light/11 hours dark cycles. To induce transgene expression, embryos, larvae and adult flies were incubated at 37 °C for 1 hour followed by a 30-minute recovery period at 25 °C. Transgenic vectors were site-specifically delivered via phiC31 system^20^ using embryo microinjection. Recipient sites for 2^nd^ and 3^rd^ chromosome integrations were strains BDSC #24482 and 24485, respectively. These two lines were arbitrarily selected as recipients for PRExpress based on the availability in the lab, absence of PcG target genes in direct vicinity^76^, absence of trapping of near-by enhancers, high survival rate after injection, light orange transformant eye color, and high transformation score^77^. Genotypes of fly lines used in this study are given in Supplementary Table 4.

### Chromatin immunoprecipitation (ChIP)

ChIP experiments on embryos collected overnight (16 hours) were performed as described previously^78^. Antibodies against Polycomb^79^, Posterior Sex Combs^80^, and H3K27me3 (Upstate, 07-449) were used for immunoprecipitations. Results of ChIP experiments were evaluated using digital droplet PCR (Bio-Rad) following manufacturer’s instructions. *bxd* sequence was quantified with primer pair 1 (Supplementary Table 5) and probe 1 (Supplementary Table 6). *Act5C* was quantified with primer pair 2 and probe 2. *poly-PRE* was quantified with primer pair 3 and probe 1. *Ubx* was quantified with primer pair 4 and probe 3. *lacZ* was quantified with primer pair 5 and probe 4. Percent of input precipitated was calculated first, these values were then normalized by percent of input precipitated of either *bxd* or *Ubx*.

### X-gal staining of embryos and larval tissues

Embryos were collected overnight (16 hours), dechorionated in 3% hypochlorite solution for 2 minutes, and washed thoroughly with water. Embryos were fixed in 33% (v/v) fixative solution (2 mM EGTA, 1 mM MgSO_4_, 7% formaldehyde, and 0.1 M Pipes, pH 6.9) in heptane, rotating at room temperature for 20 minutes. Embryos were then washed twice in PBT (0.3% Triton X-100 (v/v) in PBS, pH 7.4) and re-suspended in pre-warmed staining solution (150 mM NaCl, 1 mM MgCl_2_, 3.1 mM K_4_[Fe^II^(CN)_6_], 3.1 mM K_3_[Fe^III^(CN)_6_], 0.3% Triton X-100 (v/v), 10 mM NaH_2_PO_4_/Na_2_HPO_4_ pH 8) supplemented with 0.2% X-gal (w/v) dissolved in DMSO. The pH 8 of staining solution was used to select for LacZ protein activity compared to endogenous β-galactosidase^52, 53^. Embryos were stained for 4 hours at 37 °C. After staining, embryos were devitellinized in 1:1 solution of ethanol/methanol and vigorous shaking for 1 minute, washed 3 times with methanol, and then, gradually rehydrated by washing in 70% (v/v), 50% (v/v), 30% (v/v) methanol in PBT solution and finally in PBT.

Wandering third instar larvae (male and female) were collected and washed in PBS. Imaginal discs and larval brain were extirpated in PBS and fixed in PBS + 1% (v/v) glutaraldehyde for 20 minutes. After having been rinsed twice in PBS, discs were incubated in pre-warmed staining solution supplemented with 0.2% X-gal (w/v) dissolved in DMSO for 4 hours at 37 °C. Staining reactions were stopped by rinsing the discs in PBT. Discs were promptly imaged after staining. In all cases, tissues were over-stained for extended times to visualize also residual LacZ activity. More than 4 embryonic and larval samples were stained for each genotype. Images were acquired immediately after staining using Leica DMI6000 B microscope and Leica Application Suite X software.

### ß-galactosidase activity assay

Embryos were collected overnight (16 hours), dechorionated in 3% hypochlorite solution for 2 minutes, washed thoroughly with water, and frozen in liquid nitrogen. 20 wandering third instar larvae and six adult flies (3 days post eclosion, 3 males and 3 females), respectively, were collected per replicate and frozen in liquid nitrogen. Frozen samples were homogenized on ice in assay buffer (1 mM MgSO_4_, 2% Triton X-100 (v/v), 100 mM Hepes pH 8) supplemented with cOmplete™, EDTA-free Protease Inhibitor Cocktail (Roche). The pH 8 of assay buffer and Hepes were used to select for LacZ protein activity compared to endogenous β-galactosidase^52, 53^. Homogenates were spun twice at 15′000 rpm for 10 minutes at 4 °C, each time discarding debris and retaining supernatants. Homogenate protein concentrations were measured using Pierce BCA Protein Assay Kit (Thermo Fisher Scientific). 1 µg of protein from homogenates was incubated at 37 °C for 50 minutes in 0.1 ml of assay buffer supplemented with 4-MUG (Sigma, M1633) at 0.9 mM final concentration. ß-galactosidase converts non-fluorescent substrate 4-MUG into 4-MU, a fluorescent product. 4-MU fluorescence was measured using EnVision Multilabel Reader 2104 (PerkinElmer).

### RNA extraction, cDNA synthesis and quantitative RT-PCR

Embryos were homogenized in TRIzol Reagent (Ambion) and RNA was extracted using the Direct-zol RNA MiniPrep kit (Zymo Research) according to the manufacturer’s instructions. To remove genomic DNA contaminations, RNA samples were treated with DNase (Turbo DNA-free Kit (Ambion)). Quantification and purity of RNA was assessed on a NanoDrop Spectrophotometer (ThermoFisher Scientific). cDNA was synthesized from 1 µg RNA in a total reaction volume of 10 µL using the First Strand cDNA Synthesis Kit (ThermoFisher Scientific) as recommended by the manufacturer with the following adaptations: A 1:1 mixture of random hexamer primers and oligo(dT)_18_ primers was used and the cDNA synthesis was performed for 5 min at 25 °C followed by 60 min at 43 °C. Quantitative PCR was performed with a SYBR Green I-based reaction mix (FastStart Essential DNA Green Master (Roche Life Science)) and gene-specific primer pairs (designed using Primer3 software^81^ and NCBI Primer BLAST) (Supplementary Table 5) on a LightCycler 96 Instrument (Roche Life Science). Reaction mixtures contained 5 µL 2x FastStart Essential DNA Green Master, 1 µL primer mix (5 µM forward primer, 5 µM reverse primer) and 4 µL cDNA diluted 1:50 in nuclease-free water. qPCR conditions included a preincubation step at 95 °C for 10 min and 45 cycles of a 3-step-amplification consisting of 95 °C for 10 s, 60 °C for 10 s and 72 °C for 10 s. Expression levels of Ribosomal protein L32 (RpL32) were used for normalization. Robustly constant expression of RpL32 across samples was verified by quantification relative to a second endogenous housekeeping gene, ATPase cf6.

### Statistical analysis

The average values of three biological replicates (n = 3 for all experiments) are given as mean ± SD. Significance testing for ChIP, ß-galactosidase activity assay, and qRT-PCR experiments was performed using one-way ANOVA test. ANOVA is relatively robust for non-normally distributed data sets as well and was used throughout the study^82–84^. Shapiro-Wilk and Anderson-Darling tests^85^ were used for normality testing of data sets (Supplementary Table 7). 2 out of 27 data sets tested were non-normally distributed. Non-parametric two-sample Kolmogorov-Smirnov^86^ and Kruskal-Wallis^87^ tests were applied to these two data sets and results are reported in Supplementary Table 7. Statistical significance was defined as *P* ≤ 0.05.

## Electronic supplementary material

Supplementary Information
