# Supplementary material for: Single vector non-leaky gene expression system for Drosophila melanogaster
Source: Sci Rep. 2017 Jul 31;7:6899. doi: 10.1038/s41598-017-07282-w (PMC5537222; doi:10.1038/s41598-017-07282-w)
Supplement: Supplementary file 1 — Supplementary Information [file 41598_2017_7282_MOESM1_ESM.pdf]

Supplementary Information

Single vector non-leaky gene expression system for *Drosophila melanogaster*

Arslan Akmammedov[1], Marco Geigges[1], and Renato Paro[1,2]

[1]Department of Biosystems Science and Engineering, Federal Institute of Technology Zürich, Mattenstrasse 26, 4058 Basel, Switzerland

[2]Faculty of Science, University of Basel, Klingelbergstrasse 50, 4056 Basel, Switzerland

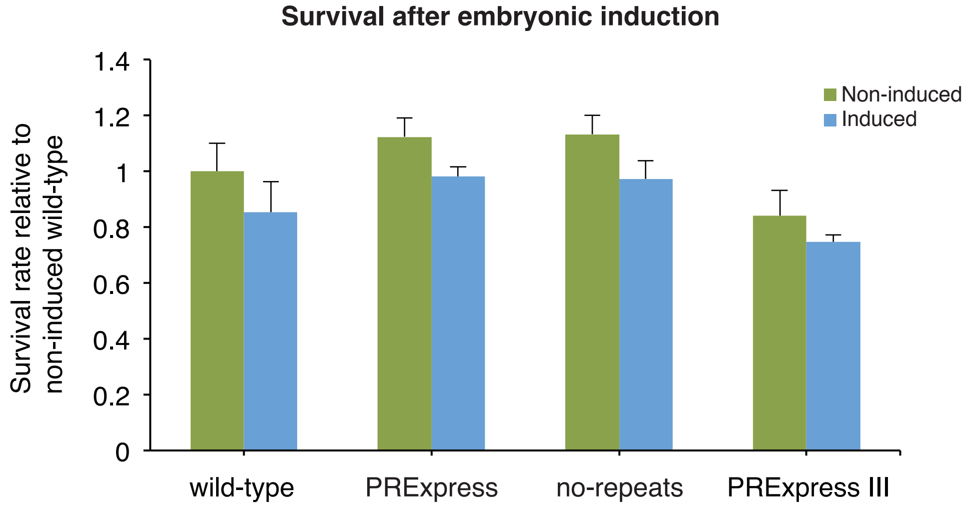

Supplementary Figure 1. Survival of embryos to adulthood after 1-hour heat-shock at the embryonic stage. Embryos were collected overnight (0-16 hours), heat-shocked for 1 hour at 37˚C, and then transferred to 25˚C. Induction at the embryonic stage reduced survival by 11-15% compared to non-induced flies in all lines tested. 50 embryos per replicate were analyzed. Wild-type and PRExpress: n = 6, no-repeats and PRExpress III: n = 2.

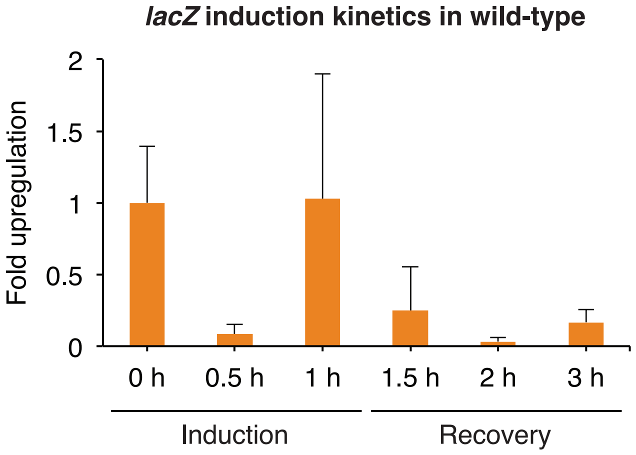

Supplementary Figure 2. Induction kinetics of *lacZ* mRNA in wild-type embryos. Since no *lacZ* gene is present in wild-type fly, this graph represents background signal using primers for *lacZ* amplification.

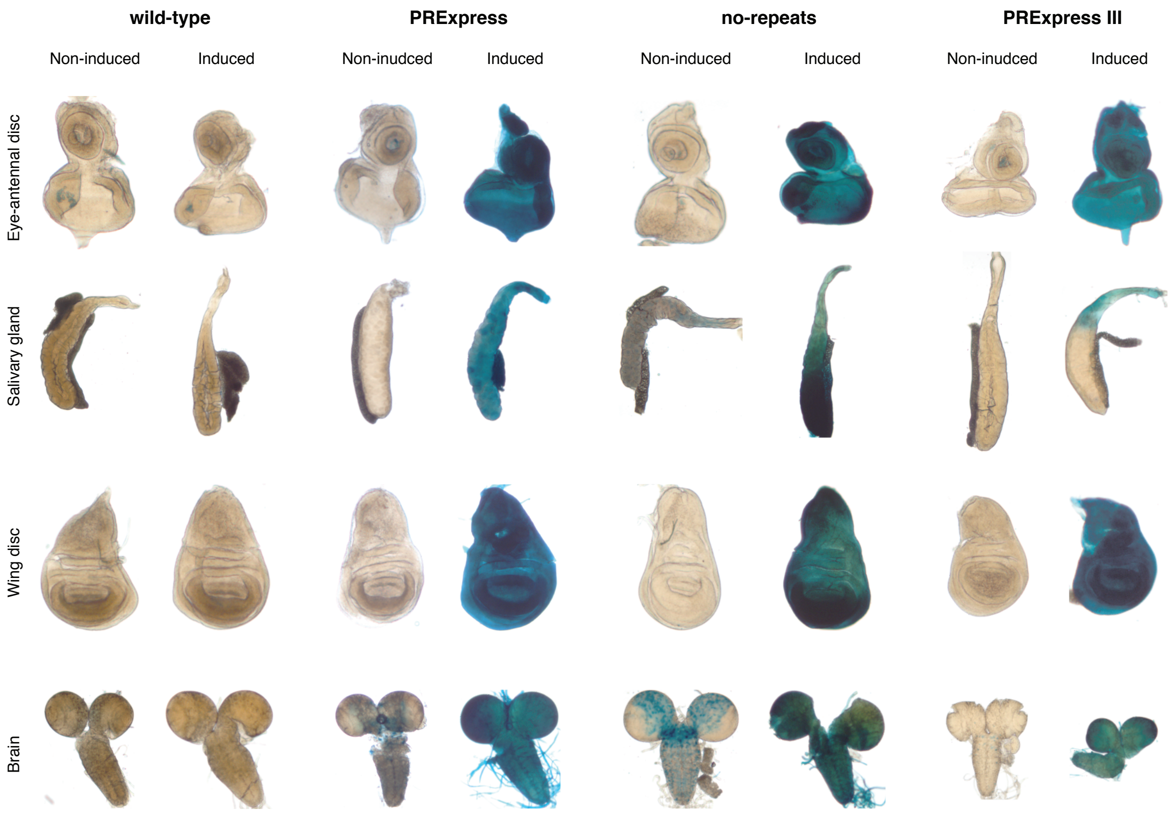

Supplementary Figure 3. X-gal staining of selected tissues from wild-type, PRExpress, no-repeats, and PRExpress III larvae. Larvae were heat shocked for 1 hour at 37˚C and allowed to recover for 30 minutes at 25˚C. After recovery period, larvae were dissected and stained.

Supplementary Table 1. Statistical significance of comparisons between non-induced and induced wild-type, PRExpress, no-repeats, and PRExpress III samples. Significance testing was performed using one-way ANOVA test.

Figure	Compared groups	*P* -values
2B	wild-type embryos	0.38
	PRExpress embryos	3.1×10^{-4}
	no-repeats embryos	3.9×10^{-9}
3A	wild-type larvae	0.64
	PRExpress larvae	1.4×10^{-4}
	no-repeats larvae	6.4×10^{-4}
3B	wild-type adults	0.22
	PRExpress adults	7.6×10^{-4}
	no-repeats adults	3.6×10^{-4}
3C	wild-type embryos	0.38
	PRExpress III embryos	2.0×10^{-10}
	wild-type larvae	0.64
	PRExpress III larvae	9.6×10^{-5}
	wild-type adults	0.22
	PRExpress III adults	4.0×10^{-3}

Supplementary Table 2. Induced *lacZ* mRNA levels in PRExpress and no-repeats lines. The values for *lacZ* mRNA were normalized to *RpL32* values and $2^{-\Delta Ct}$ values ($2^{-(Ct(lacZ)\ -\ Ct(RpL32))}$) are depicted in the table. Means of three biological replicates are shown.

Line	Time points of Fig. 2D and E					
	0 h	0.5 h	1 h	1.5 h	2 h	3 h
PRExpress	0.000126	0.127	0.174	0.181	0.0400	0.00310
no-repeats	0.00214	0.657	1.019	0.670	0.129	0.0114

Supplementary Table 3. Monomer sequences

Monomer name	5' - 3' sequence
poly-PRE monomer	tcaaactgcggtccatgtttatatagagcgtgacaccacgatgccgccataacggcagaaccaa<br>agtgccgataactcaaaaagagagagggctattccaagtctgacgtgcgtaagagcgagatac<br>agataagactacgcgcaccataatggctgcgccgtaaagcgagagcgatccgagcgagaagg<br>ctaaccgtatctctccctctctccgcagtcgcggcgcagtcgctgcctctgcagttgatcgttggg<br>aaccggagggcaaaatgtcgggcaacgatgcctgtagcaatggc
w-repeats monomer	tcaaactgcggtccatgtttatatagagcgtgacaccacgatgcctcgttgcagaatagtccgcgt<br>gtccggttgaccagctgccgccatccggagcccggctgattgaccgccccaaagatgtccatat<br>tgtgccaggcataggtgaggttctcggctagttggccgctccctgaaccggagtcctccggcgg<br>actgggtggccggagcgtgccgtagtttttggcctgcccgaagccctggttgatcgttgggaacc<br>ggagggcaaaatgtcgggcaacgatgcctgtagcaatggc
r-repeats monomer	tcaaactgcggtccatgtttatatagagcgtgacaccacgatgcctaggagtgcaccatgccgtc<br>cggagcgcccacacgcgcatgttcgccgtcgtggctgccactgatccataaccgcaggacgcg<br>aggtgctggctgttgactcgcatctagccatcttcgcgccggtgggggttgctcaggttcgagcg<br>tagccgtggccaatactggtcggtatcacttcgggcggtctcggtatcgcttgatcgttgggaacc<br>ggagggcaaaatgtcgggcaacgatgcctgtagcaatggc

Underlined are 198 bp of PRE, 2nd exon of *white* gene, and random sequences. Non-underlined are flanking spacer sequences.

Supplementary Table 4. Genotypes

Fly line name	Bloomington stock #	Genotype
wild-type	2376	Oregon-R-P2
2nd chromosome recipient	24482	y^1 M{vas-int.Dm}ZH-2A w^*; M{3xP3-RFP.attP'}ZH-51C
PRExpress		w^{1118}; M{3xP3-RFP.attR'.PRExpress.attL'}ZH-51C/M{3xP3-RFP.attR'.PRExpress.attL'}ZH-51C
no-repeats		w^{1118}; M{3xP3-RFP.attR'.no-repeats.attL'}ZH-51C/M{3xP3-RFP.attR'.no-repeats.attL'}ZH-51C
w-repeats		w^{1118}; M{3xP3-RFP.attR'.w-repeats.attL'}ZH-51C/M{3xP3-RFP.attR'.w-repeats.attL'}ZH-51C
r-repeats		w^{1118}; M{3xP3-RFP.attR'.r-repeats.attL'}ZH-51C/M{3xP3-RFP.attR'.r-repeats.attL'}ZH-51C
PRExpress in Pc^3/+		w^{1118}; M{3xP3-RFP.attR'.PRExpress.attL'}ZH-51C/M{3xP3-RFP.attR'.PRExpress.attL'}ZH-51C; Pc^3/+
PRExpress/+		w^{1118}; M{3xP3-RFP.attR'.PRExpress.attL'}ZH-51C/+
PRExpress/ $Su(var)205^5$		w^{1118}; M{3xP3-RFP.attR'.PRExpress.attL'}ZH-51C/$Su(var)205^5$
3rd chromosome recipient	24485	y^1 M{vas-int.Dm}ZH-2A w^*; M{3xP3-RFP.attP'}ZH-68E
PRExpress III		w^{1118}; M{3xP3-RFP.attR'.PRExpress.attL'}ZH-68E/M{3xP3-RFP.attR'.PRExpress.attL'}ZH-68E
UAS-lacZ	3955	w^{1118}; P{w[+mC]=UAS-lacZ.NZ}20b
UAS-lacZ	8529	w^*; P{w[+mC]=UAS-lacZ.Exel}2
QUAS-lacZ	30006	y^1 w^{1118}; P{w[+mC]=QUAS-nuclacZ.P}7
QUAS-lacZ	30007	y^1 w^{1118}; P{w[+mC]=QUAS-nuclacZ.P}44
tetO-lacZ	26804	y^1 w^*; P{w[+mC]=tetO.7-lacZ.B}2

Supplementary Table 5. Primer sequences

Primer pair	Primer target	5' - 3' sequence
1	*bxd* fwd	cgacagttatggcgacggagctgc
	bxd rev	aagagcgagatacagataagactac
2	*Act5C* fwd	ctggcattcaacattcacc
	Act5C rev	cgatttaacaaattcaaggcg
3	*poly-PRE* fwd	ctccggttcccaacgatcaactgc
	poly-PRE rev	aagagcgagatacagataagactac
4	*Ubx* fwd	cctgttatccaatccgttgc
	Ubx rev	agcgctcaaaaacaatctgg
5	*lacZ* fwd	ccaaaatcaccgccgtaagc
	lacZ rev	aggcgtttcgtcagtatccc
6	*RpL32* fwd	gacgcttcaagggacagtatctg
	RpL32 rev	aaacgcggttctgcatgag
7	*lacZ-Hsp70 3'UTR* fwd	cggtcgctaccattaccagt
	lacZ-Hsp70 3'UTR rev	tcgatcgaaacattcttatcagtct

Supplementary Table 6. Probe sequences

Probe number	Probe target	5' - 3' sequence
1	*bxd/poly-PRE*	FAM-ctaaccgtatctctccctctctcc-BHQ-1
2	*Act5C*	FAM-ccgtgcggtcgcttagctcagcctc-BHQ-1
3	*Ubx*	FAM-ccgactcaactcactcgactcggcc-BHQ-1
4	*lacZ*	FAM-cagcgactgatccacccagtcccagac-BHQ-1

Supplementary Table 7. Normality testing of data sets and non-parametric test. In the two cases with non-normal distribution, two non-parametric tests were performed to compare non-induced PRExpress to non-induced PRExpress in Pc^3 mutant background (Fig. 1C) and PRExpress larva to wild-type larva (Fig. 3A). Results of the Kruskal-Wallis test agree with the ANOVA test results, whereas results of the two-sample Kolmogorov-Smirnov test do not. Irrespective of which test results are accepted as more appropriate, the claims of the study are not changed. In case of Fig. 1C, even if the change is not significant, there is a clear trend for de-repression of the reporter in Pc^3 mutant background. In the case of Fig. 3A, even if the difference is not significant, it still suggests that PRExpress is not leaky.

Figure	Sample	Condition	Normality test		Non-parametric test	
			Shapiro-Wilk[a]	Anderson-Darling[b]	Two-sample Kolmogorov-Smirnov[a]	Kruskal-Wallis[a]
1b	PRExpress	H3K27me3 at Act5C	0.090	8.67%		
		H3K27me3 at lacZ	0.754	55.07%		
		Pc at Act5C	0.832	59.09%		
		Pc at poly-PRE	0.879	60.97%		
		Psc at Act5C	0.093	8.82%		
		Psc at poly-PRE	0.062	7.6%		
1c	PRExpress	Non-induced	0.033	6.63%	0.100	0.05
	PRExpress in Pc^3/+		0.237	15.95%		
	PRExpress	Induced	0.598	45.31%		
	PRExpress in Pc^3/+		0.349	23.82%		
	PRExpress/+	Non-induced	0.893	61.39%		
	PRExpress/ $Su(var)205^5$		0.340	23.11%		
	PRExpress/+	Induced	0.181	12.64%		
	PRExpress/ $Su(var)205^5$		0.716	53.06%		
2b	wild-type	Non-induced	1.000	63.07%		
	PRExpress		0.900	61.61%		
	no-repeats		0.176	12.4%		
2c	wild-type	mRNA	0.938	62.51%		
	PRExpress		0.664	50.63%		
	no-repeats		0.882	61.06%		
3a	wild-type	Non-induced	0.424	29.99%		
	PRExpress		0.000	5.65%	0.100	0.046
	no-repeats		0.226	15.21%		
3b	wild-type	Non-induced	0.756	55.22%		
	PRExpress		0.627	47.85%		
	no-repeats		0.331	22.44%		

3c	wild-type embryo	Non-induced	1.000	63.07%		
	PRExpress III embryo		0.454	32.53%		
	wild-type larva		0.424	29.99%		
	PRExpress III larva		0.391	27.23%		
	wild-type adult		0.756	55.22%		
	PRExpress III adult		0.085	8.47%		

a – test results are given as P -values, b – test results are given as confidence that distribution is normal.
